# The Common Forms of Dyspepsia in Women

**Published:** 1907-07-20

**Authors:** Robert Saundby

**Affiliations:** Professor of Medicine in the University of Birmingham


### Hospital Clinics.

### THE COMMON FORMS OF DYSPEPSIA IN WOMEN.

By Professor ROBERT SAUNDBY, M.D., LL.D., M.Sc., F.R.C.P., Professor of Medicine in the University of Birmingham.

I took the title of the Common forms of Dyspepsia in Women thirteen years ago for a course of lectures delivered under the conditions of the Ingleby Trust in Birmingham, because, when invited to deliver those lectures I understood that in accordance with the terms of the trust deed they were to be devoted to some subject connected with diseases of women or children, branches of practice with which the late Dr. Ingleby had been specially engaged during his life, and my mind naturally turned to the most common class of diseases of women which had come under my observation in hospital practice. For it was certainly true when I had charge of out-patients that half the women complained of "dyspepsia," that is to say, of pain or discomfort associated with taking food, and having been vividly impressed with the slight relief I could afford them by remedies such as are generally supposed to help digestive troubles, I had ultimately relied mainly on tonics, especially mineral acid tonics with strychnine or nux vomica, as affording them more relief than any other drug remedy. In order to study the subject more closely I admitted into my wards a number of such cases, and commenced their systematic study, employing for this means all the newer methods which German clinicians, especially Küssmaul and Ewald, had devised. The result was to show that a certain proportion were suffering from serious organic disease, many more from recent and more or less curable gastritis, frequently due to defective teeth, but in most the main element underlying the whole condition was debility, giving rise to atonic dilatation of the stomach, generally associated with gastroptosis. These facts had previously been pointed out by Bouchard and Glénard, but their frequent occurrence is much more generally admitted by specialists to-day than it was thirteen years ago, although they still remain virtually ignored in every-day practice. The subjects of this condition, when they come under medical treatment, usually complain of pain after food, frequently between the shoulders, of flatulence and constipation, and often present other evidences of stomach derangement, such as a furred tongue; these symptoms are treated *secundum artem* with rhubarb and soda, calomel and blue pill, which, with some good advice as to diet, may allay the recent irritation of the stomach, but the fundamental debility and atony persist. With rest in bed and careful diet, coupled with general massage, faradism, or douches to strengthen muscular tone, they improve very much, and go out cured, but such a course, to be effective, must last from two to three months, and there is always a risk of relapse owing to the unfavourable conditions under which so many women spend their lives. Generally speaking, the younger the patient the more readily recuperation takes place.

This all seems simple, but is not so easy as it looks, for, given a case of inveterate dyspepsia, it is necessary to exclude serious organic disease, which can only be done by careful examination of test meals and the use of the stomach tube. It is an advantage if the patient can be kept in bed for a few days while the necessary examinations are being made, and if there is vomiting bed is absolutely necessary for satisfactory treatment, as well as for diagnosis.

### Distension of the Stomach.

Where there is no history of recent hæmatemesis there can be no objection to proceeding with the usual means for investigating the condition of the stomach. The first of these is distension of the stomach with carbonic acid gas by giving separately in successive doses 120 grs. of bicarbonate of soda and 90 grs. of tartaric acid, dissolved in water. This is by far the most efficient means of displaying the size, shape, and position of the stomach, and having used it constantly for ten or twelve years, I am justified in saying that it is quite safe, and that it seldom causes any pain that is not promptly relieved when the patient is allowed to sit or stand up. In sparsely nourished people the outline of the distended stomach can be distinctly seen, and the great curvature and the position of the lower border determined; where there is gastroptosis the smaller curvature may also be displayed; if the outline of the distended stomach is not visible its shape and size can be approximately determined by percussion. Should the great curvature not reach to the umbilicus the size and position may be considered normal; if it reaches that point there is slight dilatation, while in more marked cases it may reach to the level of the anterior superior spines or the pubes. The size of the stomach is not always an indication of the gravity of its condition, for I have seen it dilated to the pubes in a young woman after a very long bicycle ride, yet recovery took place rapidly after a few days' rest.

### The Microscopical Examination.

Having ascertained that the stomach is dilated, and perhaps displaced downwards, the next point to determine is whether it empties itself within a reasonable time, for it may be taken as a safe rule that a normal stomach contains no remains of food twelve hours after eating. In order to determine this question the patient should take a light supper at nine o'clock, of which an essential part should be a tablespoonful of currants. Next morning at nine o'clock the stomach tube is passed, and any contents emptied by expression, or, if none can be obtained, the stomach is washed out with a little distilled water, and the washings put into a conical glass, where they are allowed to stand for microscopical examination. If any fragments of currant skin can be recognised under the microscope the dilatation depends upon pyloric obstruction, and the case is one that should be handed over to a surgeon for gastro-enterostomy.

The Test Meal.

If the microscopical examination reveals nothing but a few epithelial cells, the negative conclusion is not certain, and it may be desirable that we should empty the stomach after an eight hours' interval. In most cases it is unnecessary to make this second examination, and we proceed to examine the chemical capacity of the stomach by giving a test meal consisting of $\frac{3}{4}$ pint of weak tea without milk or sugar and $1\frac{1}{2}$ oz. or 2 oz. of bread. Exactly one hour later the stomach contents must be withdrawn by the tube, without using any washing water, and some of the resulting liquid filtered. The filtrate is examined for sugar by Fehling's solution, for erythrodextrin by iodine, and for free hydrochloric acid by Günsberg's re-agent, and its reaction to litmus paper should be noted. In normal digestion all these results should be positive. The material remaining on the filter should be examined with the microscope, and may be stained with a little weak iodine solution; where unaltered starch granules are very numerous there is probably excessive secretion of gastric juice. We may occasionally see such organisms as *sarcinæ ventriculi* and the *leptothrix* which Boas regards as associated with the presence of cancer of the stomach. It is not usually worth while to make a *quantitative* examination of the amount of free hydrochloric acid present, for the results are inconclusive. The biuret reaction should be looked for. At one time it was my regular practice to determine the chemical capacity of these stomachs by performing artificial digestive experiments with the filtered gastric contents and small pieces of hard-boiled white of egg. Four test tubes were placed in the incubator, one containing the filtered gastric contents and white of egg; the second these with the addition of pepsin; the third these with the addition of hydrochloric acid; and the fourth contained merely the egg albumen, hydrochloric acid, and pepsin used in the other experiments as a control. The result of a very large number of such experiments was to show that in these cases of atonic dyspepsia hydrochloric acid is commonly deficient, but that absence or defect of pepsin is very rare. In 138 cases free hydrochloric acid was absent in 57, deficient in 40, normal in 31, and excessive in only 10; while out of 141 cases pepsin was only absent on four occasions, and in all these hydrochloric acid was also absent. The digestion of starch is as a rule satisfactory, and is only hindered where there is an excessive secretion of gastric juice.

Some Points About Digestion.

It does not follow that in these circumstances protein digestion and assimilation are seriously affected, for the stomach defect may be made up by the action of the intestinal glands, and it is a well-established fact that cases with complete absence of active gastric juice (achylia gastrica) have presented no symptoms of impaired nutrition. This is so well known as greatly to have depreciated the reputation of the stomach as an organ of digestion, some authorities regarding it as mainly the receptacle in which salivary digestion is completed and food disinfected by the action of the hydrochloric acid of the gastric juice; as practical clinicians we know that if the food is of suitable quality, well masticated, and passed promptly out of the stomach none of the ordinary symptoms of dyspepsia arise in spite of the chemical digestive processes of the stomach being defective.

In the treament of the group of cases we are now considering we have to see how far it is possible to fulfil these conditions; namely, to provide food of suitable quality, properly masticated, or at least finely divided and mixed with saliva, and to insure its exit from the stomach in good time. The first condition is simply fulfilled, so far as the medical practitioner is concerned, as he has no great difficulty in drawing up a dietary from which are excluded all indigestible articles of food; but it is not so easy to obtain from patients cheerful and willing obedience to rules which often run counter to popular prejudices. A suitable dietary not merely excludes rich and greasy dishes, pork, salmon, pickles, pastry, port wine, etc., but must forbid porridge, brown bread, uncooked and many dried fruits, raw vegetables, all alcoholic drinks, and Indian and Ceylon teas. It is difficult to see why currant bread should be advertised in the bakers' shops on the authority of a "Physician to the King" as peculiarly digestible and nutritious, for nothing is harder to digest than the skins of currants and raisins, and although in the small quantity in which they are put into these loaves they may do no harm, when eaten in a mass, as in mincemeat and plum-pudding, they are not without reason blamed for very unpleasant consequences. Even fresh fruit which contains skins and seeds should be prepared for these patients by being put through a sieve.

Nuts are notoriously indigestible eaten at the end of a heavy dinner, yet vegetarians make a meal of them without harm. An elderly lady who suffers from atonic dyspepsia with gastric dilatation and gastroptosis has adopted a vegetarian diet with perfect success, and for some years past has obtained her protein food almost wholly from nuts. Probably one of the conditions of success is that the nuts are ground to a fine powder, for patients who have been unable to eat meat for years digest it perfectly well when given finely minced and without the addition of any other article of food, or with only a small piece of toast. The lesson to be drawn from these experiences is to simplify the diets of these patients in the first instance, and only to allow them to become complicated as health is restored. Liquids should, as a rule, only be taken two hours after eating solid food.

Mastication.

Proper mastication must be secured by preaching diligently the need for eating slowly, and if the teeth are defective the assistance of a dentist must be secured, while in the meantime the food must be finely divided by mincing or pounding or being made into purées. Mr. Horace Fletcher's experience shows how much careful mastication aids digestion and nutrition, for he has proved that a much smaller amount of food suffices when this important function is carefully performed. Hurried eating is common in the present day, and the habit does not at once produce its evil consequences, but when several masticating teeth have been lost and large gaps occur in the dental arcade, undivided lumps of food are swallowed unconsciously, and set up gastritis. The habit of rapid eating is not easily got rid of, and few patients recognise how large a part of their stomach troubles depend upon defective teeth until they are told of it. It is deplorable to see what a large number of the young women of the poorer class have no effective masticating teeth; but in men also between 50 and 60, even under favourable circumstances, the masticating efficiency of the teeth becomes greatly reduced and is a frequent cause of dyspeptic troubles.

A Note on Treatment.

It is impossible to supply the deficiency of hydrochloric acid so often observed in these cases, as the quantity required would amount to at least 2 or 3 drachms of the dilute hydrochloric acid of the British Pharmacopœia; the proposal to give 20 drop doses every twenty minutes for two hours after a meal is obviously impracticable. But small doses appear to do good when taken after a meal, possibly by inducing the opening of the pylorus, which is known to result from the presence of free hydrochloric acid in the stomach, and so facilitating the escape of the contents of the stomach into the duodenum. So long as the patient remains in the recumbent position, the weight of the food does not drag down the stomach; the great curvature remains in its normal position, and the weakened walls are assisted in their function of churning up the chyme and forcing it out of the stomach, so that the recumbent position after meals is desirable. It is doubtful whether there are any drugs which unquestionably aid this muscular action, but we are in the habit of giving strychnine, nux vomica, or caffeine with this object, and it is probable that the good effect so often experienced of a small cup of black coffee at the end of a heavy dinner is due to the dose of caffeine contained in it. While the patient is under treatment in bed the effect of faradism to the muscular walls of the abdomen should certainly be tried. My experience of intragastric faradism has been unfavourable; it is not a pleasant operation for the patient, and its beneficial results have seemed to me to be doubtful. A good deal has been published about the benefit to be derived in stomach atony from the use of high frequency currents, and no one would be more grateful than the sufferers from this condition or their medical attendants if these claims could be justified by the results. Unfortunately, none of the patients I have treated by this means derived any benefit at all from it. On the other hand, anything that improves the general health may do good; rest, change to a bracing air, freedom from anxiety and worry, particularly from household cares, favour recovery; but on the whole there is no plan of treatment which surpasses in value a complete Weir-Mitchell course with isolation, extending over three months, but with diet so modified as may be needed by the stomach condition.

With regard to general drugs such as are supposed to influence the general nervous condition, my experience is that they are disappointing. We have had during the last few years a succession of remedies, such as phosphates, glycero-phosphates and formates, or vegetable preparations of coca, kola, and the like. No doubt improvement sometimes follows the use of any remedy, but the employment of any of them in a series of cases is calculated to destroy one's belief in their real virtues. The only remedy in neurasthenic conditions which has given me striking results is the biniodide of mercury in doses of one-twelfth of a grain three times a day after food. In some cases this may be due to the presence of a syphilitic taint, for undoubtedly syphilis is a potent cause of neurasthenia, but it is worth trying even where there is nothing to suggest the presence of this cause.

